# Hypertonic saline increases lung epithelial lining fluid glutathione and thiocyanate: two protective CFTR-dependent thiols against oxidative injury

**DOI:** 10.1186/1465-9921-11-119

**Published:** 2010-08-27

**Authors:** Neal S Gould, Steve Gauthier, Chirag T Kariya, Elysia Min, Jie Huang, Day J Brian

**Affiliations:** 1 Department of Medicine, University of Colorado Denver, 12700 E 19th Ave., Aurora, CO 80045 USA; 2 Department of Immunology, University of Colorado Denver, 12700 E 19th Ave., Aurora, CO 80045 USA; 3 Department of Pharmaceutical Sciences, University of Colorado Denver, 12700 E 19th Ave., Aurora, CO 80045 USA; 4 Department of Medicine, National Jewish Health, 1400 Jackson St. Denver, CO 80206 USA; 5 Department of Immunology, National Jewish Health, 1400 Jackson St. Denver, CO 80206 USA

## Abstract

**Background:**

Cystic fibrosis is a debilitating lung disease due to mutations in the cystic fibrosis transmembrane conductance regulator protein (CFTR) and is associated with chronic infections resulting in elevated myeloperoxidase activity and generation of hypochlorous acid (HOCl). CFTR mutations lead to decreased levels of glutathione (GSH) and thiocyanate (SCN) in the epithelial lining fluid (ELF). Hypertonic saline is used to improve lung function however the mechanism is uncertain.

**Methods:**

In the present study, the effect of GSH and SCN on HOCl-mediated cell injury and their changes in the ELF after hypertonic saline nebulization in wild type (WT) and CFTR KO mice was examined. CFTR sufficient and deficient lung cells were assessed for GSH, SCN and corresponding sensitivity towards HOCl-mediated injury, in vitro.

**Results:**

CFTR (-) cells had lower extracellular levels of both GSH and SCN and were more sensitive to HOCl-mediated injury. In vivo, hypertonic saline increased ELF GSH in the WT and to a lesser extent in the CFTR KO mice but only SCN in the WT ELF. Finally, potential protective effects of GSH and SCN at concentrations found in the ELF against HOCl toxicity were examined in vitro.

**Conclusions:**

While the concentrations of GSH and SCN associated with the WT ELF protect against HOCl toxicity, those found in the CFTR KO mice were less sufficient to inhibit cell injury. These data suggests that CFTR has important roles in exporting GSH and SCN which are protective against oxidants and that hypertonic saline treatment may have beneficial effects by increasing their levels in the lung.

## Introduction

Cystic fibrosis (CF) can result in impaired pulmonary function from defects in mucus clearance resulting in chronic infections and inflammation. CF is caused by a mutation in the cystic fibrosis transmembrane regulator (*cftr*) gene leading to deficient functional capability of the CFTR transporter. CFTR was first discovered to be an apical Cl^- ^ion channel but has since been shown to be also involved in the transport of glutathione (GSH) and thiocyanate (SCN), and is responsible for maintaining levels of GSH and SCN in the epithelial lining fluid (ELF) [1-3]. The ELF hydrates the airway surfaces and provides a protective barrier against pathogens and air pollutants. The imbalance of ion transport across the lung epithelia in CF can lead to defective mucocilliary clearance which is thought to increase the risk of developing lung infections [4]. Lung pathogens can also stimulate increases in ELF GSH, whereas failure to do so in CFTR KO mice can result in increased lung inflammation and oxidative stress [5].

At least 80% of individuals with CF harbor at least one chronic bacterial pathogen, the most common of which is *Pseudomonas aeruginosa *[6]. The mucus build up in the CF lung can lead to an ideal environment for bacterial growth and makes clearance by immune cells or antibiotics difficult [7]. There is a substantial increase in neutrophil influx into the lung to fight the infection in CF. Both myeloperoxidase (MPO) and lactoperoxidase (LPO) are key enzymes involved in host defense. Both enzymes can utilize H_2_O_2 _and thiocyanate (SCN) to form the antimicrobial hypothiocyanate (HOSCN), while MPO also utilizes Cl^- ^to form hypochlorous acid (HOCl) a highly damaging oxidant [8,9]. Excess HOCl production in the lung without adequate antioxidants such as GSH and SCN can potentially lead to epithelial damage and unregulated inflammatory responses.

While there is no cure for CF, there are therapies that have shown some beneficial effects in clinical studies. One therapy is the nebulization of hypertonic saline for improving lung function in CF individuals [10,11]. The hypothesis is that hypertonic saline is beneficial for the hydration of the ELF which aids in mucocilliary clearance [4]. In addition to CF, hypertonic saline inhalation has been suggested for use in chronic obstructive pulmonary disease (COPD), asthma, and even pneumonia [12-14]. In the present study, the effect of hypertonic saline treatment on both GSH and SCN in the ELF was examined. In addition, the differential levels of GSH and SCN in CFTR sufficient and deficient cells was characterized and the corresponding sensitivity to HOCl mediated cell death was examined. Finally the relative increases in antioxidant concentrations observed with hypertonic saline was tested to see if it could provide further protection against HOCl mediated toxicity.

## Materials and methods

### Chemicals

All chemicals and enzymes were purchased from Sigma-Aldrich with the exception of Myeloperoxidase (MPO) which was purchased from Calbiochem.

### Cell culture

Human lung bronchial epithelial cells either sufficient (C38) or deficient (IB3) in CFTR expression or A549 cells were used and obtained from the American Type Culture Collection (ATCC) [15]. The C38 cell line is derived from the IB3 cell line with normal human CFTR stably transfected. The A549 cells were utilized as a control non-transfected airway cell line. C38 and IB3 cells were cultured in a transwell system at an air-liquid interface using LHC-8 medium containing 10% fetal bovine serum with antibiotics. A549 cells were cultured in Ham's F-12 medium supplemented with 10% FBS and antibiotics. Cytotoxicity was mesaured by LDH released in media. The % LDH release was calculated as [LDH_(media)_/LDH_(total)_] × 100 as previously described [16].

### MPO Enzyme System

The enzyme system contains three components; MPO, glucose, and glucose oxidase. Glucose and glucose oxidase were used to generate H_2_O_2_. The full enzyme system was slightly modified [17], with final concentrations of 3 U MPO, 40 mU glucose oxidase, and 100 mM glucose added to sterile PBS. Cells were incubated for 2 and 4 hours, after which the PBS was replaced with fresh medium and cytotoxicity was measured 24 hours later.

### Hypochlorite System

The stock concentration of sodium hypochlorite (HOCl) was determined based on the absorbance at 290 nm (pH 12, ∈ = 350 M^-1 ^cm^-1^) and diluted to the necessary concentration in water. A549 cells were exposed to HOCl with or without SCN and GSH in PBS for 5 minutes. PBS was then removed and replaced with fresh media. Cytotoxicity was analyzed 24 hours later by LDH release.

### Animals

Adult wild-type male C57BL/6 mice were purchased from Jackson Laboratories (Bar Harbor, ME). C57BL/6J congenic gut-corrected *Cftr *KO-Tg mice that possess a S489X truncated mutation in the murine equivalent to CFTR and have intestinal specific expression of normal human *Cftr *driven by the fatty acid binding promoter were originally obtained from Case Western Reserve University's CF Animal Core, as previously described [18]. Mice were anesthetized and exsanguinated by cardiac puncture. Plasma was collected to determine bronchoalveolar lavage (BAL) dilution factor by urea analysis (Teco Diagnostics) [16]. BAL was performed by cannulating the trachea and two 750 μL aliquots of isotonic potassium phosphate buffer (50 mM) at a pH of 7.4 were instilled and collected by gentle aspiration. Animal studies were approved by the National Jewish Health Animal Care and Use Committee.

### Hypertonic saline preparation and nebulization

Hypertonic saline was made by adding NaCl to PBS to the desired final concentration of 7% and sterile filtered. A Plexiglas nebulizing chamber was utilized with dimensions of 21.5 × 14 × 12 cm (L × H × W). Five mice at a time were kept in the chamber and exposed to aerosolized isotonic (0.9%) or hypertonic saline (7%) for 30 minutes using a DeVilbiss pulmosonic nebulizer (DeVilbiss). Normal or hypertonic saline was freshly prepared and up to 10 mL was nebulized over the 30 minute exposure. Mice were sacrificed 2 hours following saline administration and lavaged for ELF GSH and SCN determinations.

### Measurement of Glutathione

Total glutathione (GSH) was analyzed spectrophotometrically in the BALF, plasma, BAL cells, and lung tissue as previously described [19]. GSH was measured by adding the standard or sample to 100 μL of a 1:1 mixture of 3 units/mL GR with 0.67 mg/mL 5,5'-Dithiobis(2-nitrobenzoic acid) (DTNB). The reaction was initiated by the addition of 20 μL of 0.67 mg/mL NADPH and the increase in absorbance at 450 nm was monitored. For GSSG measurements the samples were incubated with 4-vinyl pyridine for 1 h to conjugate any reduced GSH prior to analysis, GSSG samples were analyzed the same way as GSH samples. The limits of detection were 0.2 μM for both GSH and GSSG. Apical GSH in cell culture was quantified using HPLC with fluorescence detection as previously described [16]. For analysis using HPLC, the GSH was first derivatized to monobromobimane for 30 min, the reaction was stopped by acidification using perchloric acid and any precipitate was removed by centrifugation. The fluorescent detector was set at an excitation and emission wavelength of 390 and 480 nm. The limit of detection is 0.1 μM.

### Measurement of Thiocyanate

Thiocyanate (SCN) was measured spectrophotometrically in BALF, lung tissue, and media with slight modifications [20]. 100 μL standard or sample was added to a microcentrifuge tube and a final concentration of 5% trichloroacetic acid (TCA) was added. The standards or samples were then transferred to a 96-well plate in duplicate with 50 μL per well and 50 μL chlorinating reagent (197 mM Na_2_HPO_4 _and 0.6% (v/v) NaOCl) was added. After which, 30 μL colorimetric reagent (463 mM NaOH, 224 mM 1,3-dimethylbarbituric acid, 232 mM isonicotinic acid) was added. The plate was placed in the dark to incubate for 5 min, after which the absorbance at 607 nm was determined using a SpectraMax 340PC plate reader. The sample concentration was based on an 8 point standard curve made from known concentrations of NaSCN.

### Statistical analysis

Data are expressed as the mean ± standard error of the mean. Prizm version 5 (GraphPad, San Diego, CA) was used to perform one or two way ANOVA statistical analysis with a Bonferroni multiple comparison test. A p < 0.05 was considered significant.

## Results

### Hypochlorous (HOCl) elicits comparable toxicity to MPO

**Figure 1:**
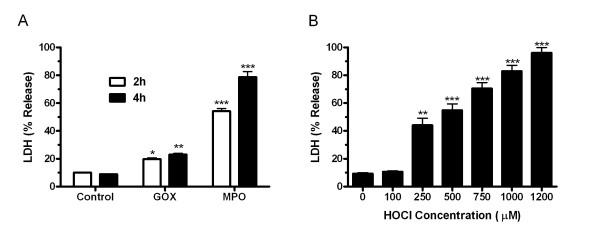
**HOCl elicits comparable toxicity to MPO**. A549 cells were exposed to MPO for 2 and 4 hours (A) or HOCl for 5 minutes (B) and toxicity was compared 24 hours after the exposure. MPO requires H_2_O_2 _to catalyze the formation of HOCl, therefore the glucose/glucose oxidase (GOX) system was utilized to produce H_2_O_2_. The MPO/GOX showed significant increases in toxicity at both 2 and 4 hour exposures. In comparison, HOCl alone produced a concentration dependant increase in toxicity. Roughly 750 μM HOCl exhibited similar toxicity as MPO at 2 hours. Data presented as mean ± SEM, *p ≤ 0.05, **p ≤ 0.01, ***p ≤ 0.001 compared to controls.

MPO is involved in host defense and is released by neutrophils to help fight bacterial infections. MPO can utilize H_2_O_2 _and Cl^- ^to produce HOCl, a highly damaging oxidant. The toxicity of a complete MPO system was tested with 2 and 4 hour exposures in lung A549 cells (Fig 1A). Glucose and glucose oxidase was used to generate H_2_O_2_, resulting in increased toxicity by itself. When MPO was added, the toxicity was significantly increased over the control levels at both 2 and 4 hours. To establish a relative concentration of HOCl that elicits the same level of toxicity as the MPO system, A549 cells were exposed to HOCl in PBS for 5 min at varying concentrations (Fig 1B). Cell toxicity increased in a concentration dependant manner with HOCl. The concentration of HOCl resulting in 100% toxicity was 1200 μM HOCl, whereas the lower HOCl concentration of 750 μM produced roughly 65% toxicity and was comparable to the MPO after a 2 hour exposure.

### CFTR deficiency increases sensitivity to HOCl mediated toxicity

**Figure 2:**
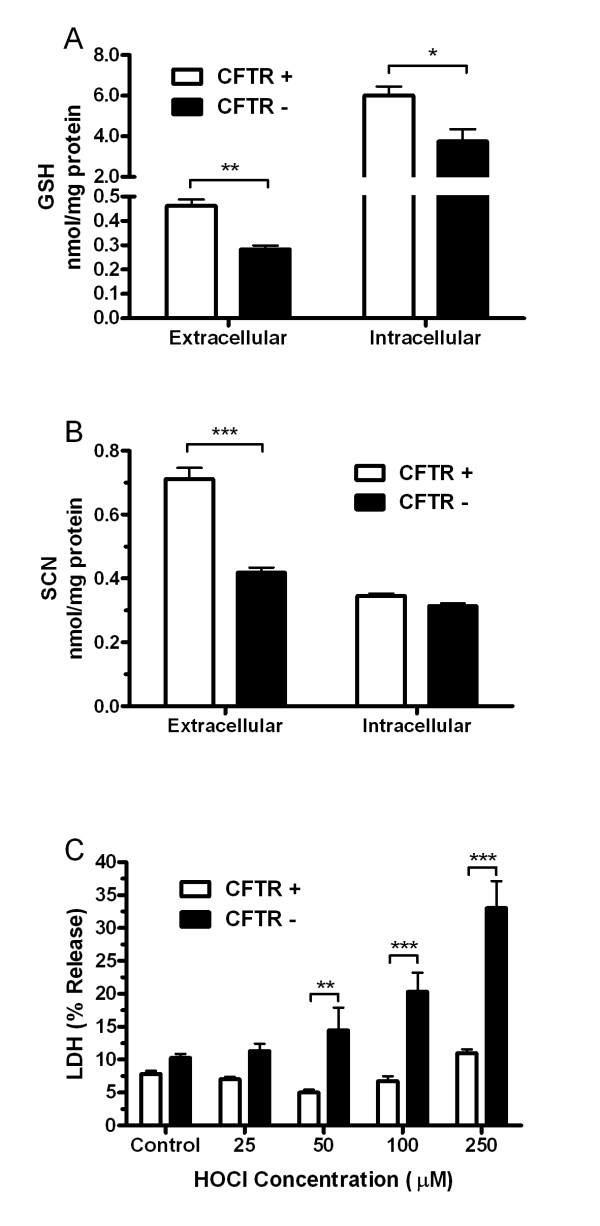
**Cftr deficiency sensitizes cells to HOCl mediated toxicity**. The levels of both extracellular and intracellular GSH (A) and SCN (B) were examined in cells either sufficient (C38) or deficient (IB3) in CFTR grown on an air liquid interface. In addition, both the CFTR sufficient and deficient cells were treated HOCl (C) to examine cell sensitivity. Data presented as mean ± SEM, *p ≤ 0.05, **p ≤ 0.01, ***p ≤ 0.001 compared between CFTR (+) and CFTR (-)

The ability to maintain elevated levels of antioxidants in the extracellular space is critical since the MPO and the corresponding HOCl that is generated are largely extracellular. CFTR is responsible for maintaining GSH and SCN, both important antioxidants involved in the detoxification of HOCl. GSH (Fig 2A) and SCN (Fig 2B) were measured in the extracellular and intracellular compartments of both CFTR (+) and CFTR (-) cell lines grown on an air liquid interface. Basal extracellular GSH and SCN levels in the CFTR (-) cell lines were roughly half of the CFTR (+) cell line. To determine if the decrease in antioxidant capacity in the CFTR (-) cell line results in increased sensitivity to oxidants, both cell lines were treated with HOCl at increasing concentrations (Fig 2C). While the HOCl concentrations between 50 and 250 μM were not toxic for the CFTR (+) cells, the CFTR (-) cells exhibited a 3-fold increase in HOCl mediated toxicity at 250 μM showing that the CFTR (-) cells are more sensitive to HOCl mediated toxicity.

### Hypertonic saline increases ELF GSH and SCN and is dependant on CFTR

**Figure 3:**
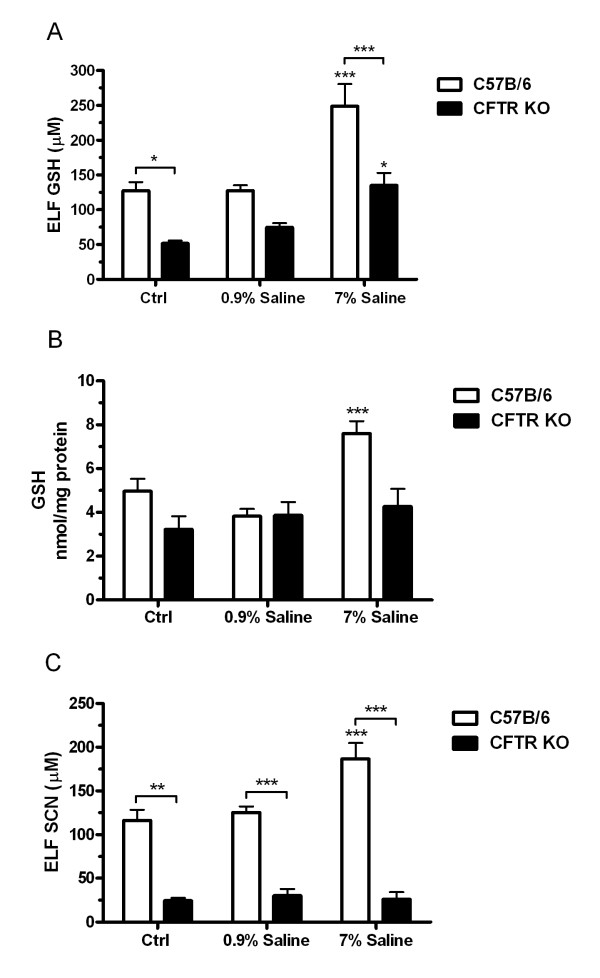
**Hypertonic saline exposure increases levels of antioxidant thiols in the ELF**. Either WT or gut corrected CFTR KO mice were exposed to hypertonic (7%) or isotonic (0.9%) saline for 30 minutes and the antioxidant response was examined in the ELF 2 hours later. Hypertonic saline increases ELF GSH (A) in both the WT and CFTR KO mice, but the CFTR KO levels only increased back up to basal levels of the WT mice. Hypertonic saline also increases the BAL cell GSH (B) in the WT but not CFTR KO mice. Finally, hypertonic saline increases ELF SCN (C) in the WT mice but not the CFTR KO mice. Data presented as mean ± SEM, *p ≤ 0.05, **p ≤ 0.01, ***p ≤ 0.001 compared to respective unexposed control or between WT and CFTR KO.

The inhalation of hypertonic saline is one therapy for CF, and these studies sought to determine if this therapy has any beneficial effects at increasing GSH or SCN in the airways. Both CFTR KO and WT mice were either exposed to nebulized isotonic (0.9%) or hypertonic (7%) saline, to control for potential effects of isotonic saline, unexposed mice were also evaluated. The CFTR KO mice had roughly half the basal ELF GSH levels as compared to the WT mice, with levels only reaching 51 μM GSH compared to 127 μM GSH (Fig 3A). While the isotonic saline had no effect on WT ELF GSH, the CFTR KO mice did exhibit a small increase in GSH up to 74 μM. Conversely, WT mice exposed to hypertonic saline had roughly twice the amount of ELF GSH with levels reaching 248 μM compared to only 123 μM in the CFTR KO mice. In addition, BAL cell GSH levels were increased in the WT mice but not in the CFTR KO mice with hypertonic saline exposure (Fig 3B).

While GSH is a primary antioxidant in the ELF, SCN is also an important antioxidant at detoxifying HOCl from excess MPO activity and is dependent on CFTR to be exported into the ELF. ELF SCN levels were measured in both the WT and CFTR KO mice after exposure to either isotonic or hypertonic saline (Fig 3C). The WT mice did show an increase in ELF SCN with the hypertonic saline exposure, while the CFTR KO mice did not exhibit increases in SCN. This suggests that while hypertonic saline may afford some protection by increasing GSH in CFTR KO airways, this protective response is limited by the inability to elevate their already deficient ELF SCN levels.

### Hypertonic saline does not affect lung tissue levels of GSH or SCN

**Figure 4:**
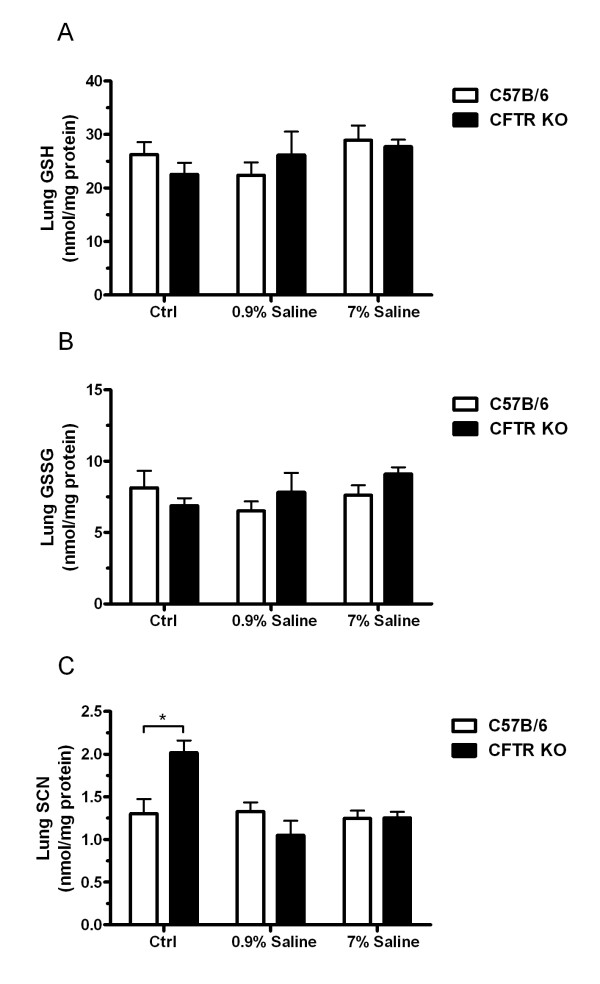
**Hypertonic saline does not affect tissue levels of GSH or SCN**. Since hypertonic saline can produce a mild osmotic stress on the lung the levels GSH (A) and GSSG (B) were examined in the lung tissue. Hypertonic saline did not alter the tissue levels of either GSH or GSSG in the WT or CFTR KO mice. In addition, tissue SCN levels (C) were examined and found to be higher in the CFTR KO unexposed lung compared to the WT, but neither hypertonic nor isotonic saline showed any effect on these levels. Data presented as mean ± SEM, *p ≤ 0.05 compared between WT and CFTR KO.

Since hypertonic saline does increase the ELF levels of both GSH and SCN in WT mice, the lung tissue GSH and SCN levels were measured to determine whether the intracellular levels are also increased. Hypertonic saline did not have any effect on either intracellular GSH (Fig 4A) or GSSG (Fig 4B). To confirm that the intracellular GSH steady-state levels were not changing the rate limiting enzyme in GSH synthesis, γ-glutamylcysteine ligase (GCL), was examined and no increases in GCL expression were found (data not shown).

CFTR is one of the main apical transporters of SCN in the lung; therefore studies to determine whether intracellular SCN was higher in the CFTR KO mice compared to the WT mice were conducted (Fig 4C). Intracellular SCN was roughly 35% higher in the unexposed CFTR KO mice compared to the WT. While there were no differences between CFTR KO and WT with both isotonic and hypertonic saline exposure.

### The concentrations of GSH and SCN achieved by hypertonic saline protect cells against HOCl mediated toxicity

**Figure 5:**
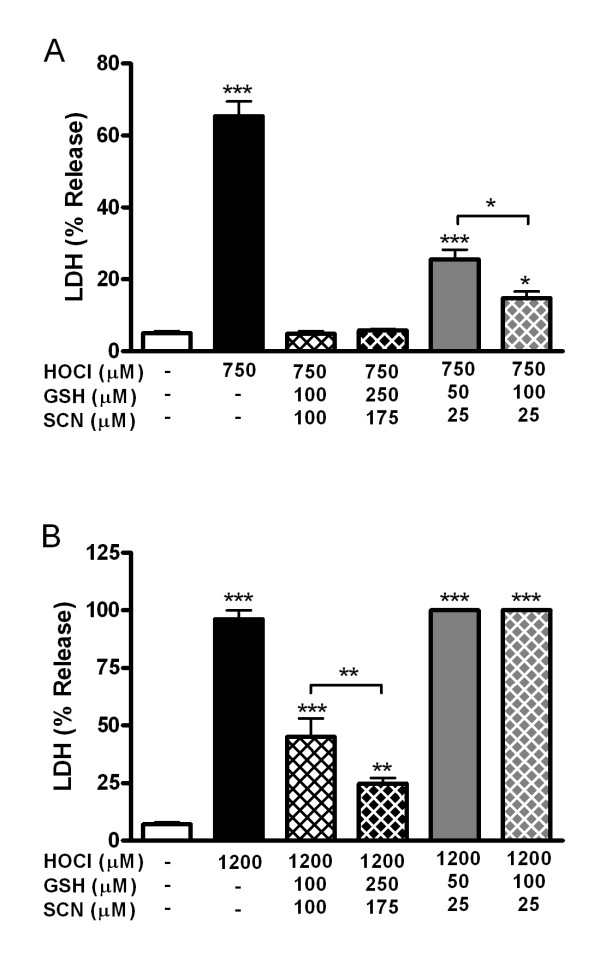
**GSH and SCN levels found in the ELF are sufficient to protect against HOCl mediated toxicity in vitro**. A549 cells were exposed to 750 μM HOCl (A) or 1200 μM (B) for 5 minutes and various concentrations of GSH and SCN were used in combination to determine if they were sufficient to provide protection against HOCl mediated toxicity assessed by %LDH release 24 hour after HOCl exposure. The combination of 100 μM GSH and 100 μM SCN represents basal levels found in the unexposed WT ELF, 250 μM GSH and 175 μM SCN represents WT hypertonic saline exposed levels, 50 μM GSH and 25 μM SCN represents CFTR KO unexposed basal levels, and 100 μM GSH and 25 μM SCN represents CFTR KO hypertonic saline exposed levels. Data presented as mean ± SEM, *p ≤ 0.05, ***p ≤ 0.001 compared to control.

Since hypertonic saline is able to increase ELF GSH but not SCN, studies to determine whether this could have significant implications for the detoxification of HOCl were conducted (Fig 5). The same concentrations of GSH and SCN as seen in the ELF were used in vitro to assess their combined effects on the toxicity of HOCl. To replicate the concentrations in the ELF, four different combinations of GSH and SCN were used. To represent the basal WT ELF levels, 100 μM GSH and 100 μM SCN were used in combination. To represent the WT ELF levels after hypertonic saline exposure, 250 μM GSH and 175 μM SCN was used. To represent the basal CFTR KO ELF levels, 50 μM GSH and 25 μM SCN was used. Finally, to represent the CFTR KO levels after hypertonic saline exposure, 100 μM GSH and 25 μM SCN was used. 750 μM HOCl alone produced roughly 65% toxicity while both combinations of GSH and SCN that represent the WT ELF exhibited complete protection against this level of HOCl-mediated injury (Fig 5A). In addition, while there was still some protection afforded by the concentrations of GSH and SCN found in the CFTR KO ELF, HOCl mediated toxicity was significantly higher than representative WT levels. The 50 μM GSH and 25 μM SCN levels associated with CFTR KO basal ELF showed roughly 25% LDH release, while the concentrations that represent the CFTR KO ELF exposed to hypertonic saline did significantly decrease the HOCl mediated toxicity at roughly 15% toxicity, which was still higher than the control. To examine whether protective effects occurred at higher HOCl concentrations, 1200 μM HOCl was used (Fig 5B). HOCl (1200 μM) alone produced nearly 100% toxicity and no protection was observed for the levels of GSH and SCN that represent the CFTR KO ELF. Conversely the levels of GSH and SCN that represent the WT ELF showed good protection with 100 μM GSH and SCN having 45% LDH release while 250 μM GSH and 175 μM SCN decreased the toxicity of HOCl to only 25% LDH release. These data suggest that there is a large dynamic range of protection against HOCl from the GSH and SCN levels associated with the WT ELF but a much smaller degree of protection in the GSH and SCN levels associated with the CFTR KO ELF.

## Discussion

**Figure 6:**
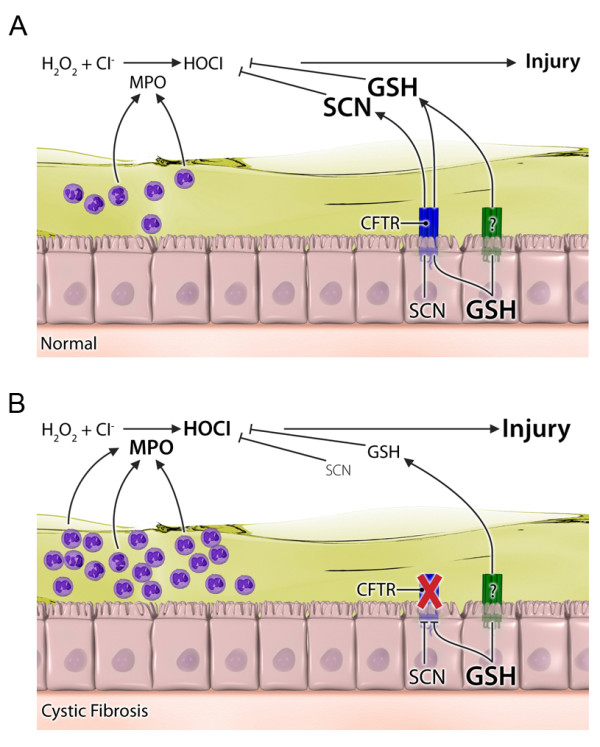
**Illustration depicting the potential role of hypertonic saline mediated changes in GSH and SCN levels and protection against HOCl mediated lung injury**. The normal airways (A) have the ability to export large amounts of both GSH and SCN into the ELF through CFTR and an unknown transporter to detoxify the relatively low amount of HOCl produced from neutrophil derived MPO. Whereas in the CF airways (B) there is a large excess of MPO producing higher amounts of HOCl. Consequently the deficiency in CFTR leads to lower levels of both GSH and SCN in the ELF which is not fully sufficient at protecting against lung injury.

A defining characteristic of CF is exaggerated inflammation and oxidative stress in the lungs of individuals with this fatal genetic disease [21]. A large fraction of individuals with CF harbor chronic infections with pathogens such as *Pseudomonas aeruginosa*, which thrive in the excess mucus secretions found in CF airways [7]. This excess mucus not only facilitates bacterial growth but it also makes it difficult for the immune response to eradicate the infection. This constant battle between the chronic pathogen exposure and immune response is a contributing factor to the excessive inflammation seen in the CF airways. Along with increases in inflammatory cytokines in the airways, there is an excessive neutrophil infiltration that is a major source of MPO. MPO utilizes H_2_O_2 _and Cl^- ^to form HOCl, a highly damaging oxidant that has known antibacterial properties [9]. Under normal circumstances the lung is well protected by its antioxidant defenses, yet the lack of CFTR impairs the lung's ability to maintain high levels of antioxidants in the ELF which could eventually result in lung damage (Fig 6) [5].

Although GSH is the main extracellular antioxidant exported by lung epithelium, SCN is also exported and can act as an antioxidant [22]. SCN can compete with Cl^- ^in the MPO catalyzed reactions to form HOSCN, an antimicrobial that is much less damaging than HOCl [23]. In this sense SCN is not necessarily acting as an antioxidant, but our lab and others have shown the antioxidant properties of SCN by directly reacting with HOCl to detoxify it (unpublished data) [24]. In the present study, the toxicity of both MPO and HOCl alone were compared and found that 750 μM HOCl elicits comparable toxicity to the MPO system. The total amount of MPO used in the in vitro systems is comparable to the MPO activity measured in lung infection models as well as isolated sputum from CF patients [25,26]. Unfortunately direct measurement of HOCl in the airways has not been done and would be difficult since HOCl is quite reactive. Based on MPO activities from sputum of CF patients, HOCl concentrations have been estimated to average 2.65 mM [27]. While the actual levels of HOCl that MPO generates in the airways is not known, it is conceivable that in isolated microenvironments the concentration of HOCl could easily reach at least 750 μM during infection or disease. In addition, the use of HOCl alone excluded any direct effects that either GSH or SCN may have on the MPO system or toxicity that may occur due to H_2_O_2_.

CFTR is currently the only known apical transporter in the lung epithelium that maintains GSH levels in the ELF [1]. As such, a deficiency in CFTR leads to decreased extracellular GSH and impairs GSH export, which is an important lung adaptive response to environmental agents. SCN has also been shown to be transported through CFTR, however the exact mechanism of how this occurs is still poorly understood [22]. The current study shows that an in vitro and in vivo deficiency in CFTR leads to lower levels of extracellular GSH and SCN. In addition, these studies show the lack of extracellular GSH and SCN may sensitize CFTR deficient cells to HOCl mediated toxicity. Although the CFTR KO mouse utilized in this study does not develop spontaneous lung disease, it is an ideal model system to examine the effects of CFTR transport deficiency without the confounding effects of lung damage and excess inflammation.

Nebulized hypertonic saline has been used for years as a sputum inducing agent in not only CF but also COPD and asthma [10,28,29]. Recently the effects of hypertonic saline in CF individuals have shown marked improvements in lung function [30]. The improvements in lung function due to hypertonic saline are currently contributed to airway fluid hydration but the exact reason is not well understood. Mutations in CFTR result in decreased fluid secretions and an increased fluid reabsorption leading to mucus dehydration [31]. Inhalation of hypertonic saline creates an osmotic gradient that pulls water into the airways, effectively hydrating the ELF, however the current studies would also suggest that hypertonic saline also increases extracellular levels of GSH and SCN. Improved mucus clearance, lung function, and reduced exacerbations have been linked to hypertonic saline treatment without serious adverse effects [4,10].

The source of SCN is commonly thought to be mainly dietary or as a metabolite of cyanide detoxification catalyzed by rhodenase [32,33]. SCN seems to be highly regulated in that it is kept at high concentrations in the extracellular space in much the same manner as GSH. It is unlike GSH in that the intracellular levels of SCN are lower in the intracellular compartment than in the extracellular space. The current studies suggest that the increase in SCN in the ELF with hypertonic saline is most likely not due to any differences in dietary uptake, which would indicate that the lung epithelium posses the ability to synthesize SCN or that there is a pool of readily available SCN for release. This point was best illustrated in cultured cells where a dietary source could not account for the differences in SCN levels. Secondarily in the CFTR KO mice there is an increase in SCN in the lung tissue of the unexposed mice compared to the WT mice, but upon either isotonic or hypertonic saline exposure the intracellular SCN returns to the normal WT levels. There could be a number of explanations for this occurrence, but it is possible that the hypertonic saline induces a recycling pathway for the excess SCN to be utilized for the synthesis of other compounds, possibly even GSH, when it can not be exported through CFTR. Unfortunately there is not much known about the regulation of SCN production and it requires further investigation.

While CFTR may be one of the only known apical transporters of GSH in the lung, the present studies show that there is still an increase in ELF GSH with the hypertonic saline treatment in the CFTR KO mice, although not nearly to the same degree as the WT suggesting other apical GSH transporters. The current studies show that even small increases in ELF GSH observed in the hypertonic exposed CFTR KO mice can still have protective effects against HOCl mediated toxicity, although not nearly to the same degree as occurs in the WT ELF. There is also previous work to suggest that osmotic stress can cause the maturation of CFTR, allowing it to reach the plasma membrane [34]. Hypertonic saline produces a transient osmotic stress and investigators have shown that osmotic agents such as sorbitol and myo-inositiol can cause maturation of ΔF508 CFTR, a trafficking mutation, leading to functional CFTR at the plasma membrane [35]. While the ΔF508 mutant may be the most common mutation of CFTR, it is by no means the only one. The CFTR KO mice utilized in this study possess the S489X truncation mutation resulting in a non functional CFTR. The present studies show that despite having a non functional CFTR one can increase ELF GSH with hypertonic saline exposure. This would indicate that anyone with CF, independent of the type of mutation, may benefit from hypertonic saline treatment yet those who still have the functional ΔF508 mutation may benefit the most.

In contrast to GSH, the effect that hypertonic saline has on SCN is clearly heavily dependent on functional CFTR. Again, CF individuals carrying the ΔF508 trafficking mutation may benefit more from hypertonic saline than those without non-trafficking CFTR mutation. The present study demonstrates that ELF SCN is only increased in the WT but not the CFTR KO mice when exposed to hypertonic saline. This would suggest that while hypertonic saline may be beneficial for some antioxidants like GSH in CF, it doesn't increase others like SCN. In addition, since SCN is involved in the MPO mediated production of HOSCN, an antimicrobial, hypertonic saline may help with the host defense protection. This would suggest that hypertonic saline may be beneficial in other inflammatory lung diseases where there is functional CFTR, like COPD or asthma. Although there is some indication that hypertonic saline may not work for advanced stages of COPD, it may be beneficial for those with early stage, or mild COPD [36]. There are numerous lung diseases that cause excessive oxidative stress and inflammation where the ability to increase both GSH and SCN with hypertonic saline treatment may be beneficial. While currently there are no studies that have shown a beneficial effect of hypertonic saline in diseases other than CF, the present study warrants further investigation of the potential mechanisms of this currently available beneficial therapy in other lung diseases.

## Conclusions

Hypertonic saline, a common therapeutic for CF, induces an increase in both GSH and SCN in the ELF. While hypertonic saline does increase GSH in CFTR KO mice, SCN is much more dependent on CFTR for export. The increases in both GSH and SCN from can protect airway epithelium from HOCl mediated injury. These findings indicate another therapeutic benefit of hypertonic saline, apart from hydration of the airways, is through increasing airway antioxidant levels and protecting against endogenously formed oxidants.

## Competing interests

The authors declare that they have no competing interests.

## Authors' contributions

NSG wrote the manuscript and performed the experiments and analyzed the samples. EM helped with obtaining in vivo samples and processing. SG, CK, and JH performed sample analysis. BJD conceived the study and wrote the manuscript. All authors have read and accepted the final manuscript

## References

[B1] Gao L Kim KJ Yankaskas JR Forman HJ Abnormal glutathione transport in cystic fibrosis airway epithelia Am J Physiol Lung Cell Mol Physiol 1999 277 1 Pt 1 L113 118 10.1152/ajplung.1999.277.1.L113 10409237

[B2] Kogan I Ramjeesingh M Li C Kidd JF Wang Y Leslie EM Cole SP Bear CE CFTR directly mediates nucleotide-regulated glutathione flux Embo J 2003 22 9 1981 1989 10.1093/emboj/cdg194 12727866 PMC156066

[B3] Conner GE Wijkstrom-Frei C Randell SH Fernandez VE Salathe M The lactoperoxidase system links anion transport to host defense in cystic fibrosis FEBS Lett 2007 581 2 271 278 10.1016/j.febslet.2006.12.025 17204267 PMC1851694

[B4] Riedler J Reade T Button B Robertson CF Inhaled hypertonic saline increases sputum expectoration in cystic fibrosis J Paediatr Child Health 1996 32 1 48 50 10.1111/j.1440-1754.1996.tb01541.x 8652214

[B5] Day BJ van Heeckeren AM Min E Velsor LW Role for cystic fibrosis transmembrane conductance regulator protein in a glutathione response to bronchopulmonary pseudomonas infection Infect Immun 2004 72 4 2045 2051 10.1128/IAI.72.4.2045-2051.2004 15039325 PMC375208

[B6] Lyczak JB Cannon CL Pier GB Lung infections associated with cystic fibrosis Clin Microbiol Rev 2002 15 2 194 222 10.1128/CMR.15.2.194-222.2002 11932230 PMC118069

[B7] George AM Jones PM Middleton PG Cystic fibrosis infections: treatment strategies and prospects FEMS Microbiol Lett 2009 300 2 153 164 10.1111/j.1574-6968.2009.01704.x 19674113

[B8] El-Chemaly S Salathe M Baier S Conner GE Forteza R Hydrogen peroxide-scavenging properties of normal human airway secretions Am J Respir Crit Care Med 2003 167 3 425 430 10.1164/rccm.200206-531OC 12446267

[B9] Klebanoff SJ Myeloperoxidase: friend and foe J Leukoc Biol 2005 77 5 598 625 10.1189/jlb.1204697 15689384

[B10] Eng PA Morton J Douglass JA Riedler J Wilson J Robertson CF Short-term efficacy of ultrasonically nebulized hypertonic saline in cystic fibrosis Pediatr Pulmonol 1996 21 2 77 83 10.1002/(SICI)1099-0496(199602)21:2<77::AID-PPUL3>3.0.CO;2-M 8882210

[B11] Fuchs HJ Borowitz DS Christiansen DH Morris EM Nash ML Ramsey BW Rosenstein BJ Smith AL Wohl ME Effect of aerosolized recombinant human DNase on exacerbations of respiratory symptoms and on pulmonary function in patients with cystic fibrosis. The Pulmozyme Study Group N Engl J Med 1994 331 10 637 642 10.1056/NEJM199409083311003 7503821

[B12] Wilson AM Leigh R Hargreave FE Pizzichini MM Pizzichini E Safety of sputum induction in moderate-to-severe smoking-related chronic obstructive pulmonary disease Copd 2006 3 2 89 93 10.1080/15412550600651339 17175671

[B13] Bathoorn E Liesker J Postma D Koeter G van Oosterhout AJ Kerstjens HA Safety of sputum induction during exacerbations of COPD Chest 2007 131 2 432 438 10.1378/chest.06-2216 17296644

[B14] Fahy JV Boushey HA Lazarus SC Mauger EA Cherniack RM Chinchilli VM Craig TJ Drazen JM Ford JG Fish JE Safety and reproducibility of sputum induction in asthmatic subjects in a multicenter study Am J Respir Crit Care Med 2001 163 6 1470 1475 11371420 10.1164/ajrccm.163.6.9901105

[B15] Flotte TR Afione SA Solow R Drumm ML Markakis D Guggino WB Zeitlin PL Carter BJ Expression of the cystic fibrosis transmembrane conductance regulator from a novel adeno-associated virus promoter J Biol Chem 1993 268 5 3781 3790 7679117

[B16] Kariya C Chu HW Huang J Leitner H Martin RJ Day BJ Mycoplasma pneumoniae infection and environmental tobacco smoke inhibit lung glutathione adaptive responses and increase oxidative stress Infect Immun 2008 76 10 4455 4462 10.1128/IAI.00136-08 18644874 PMC2546817

[B17] Bosch EH van Doorne H de Vries S The lactoperoxidase system: the influence of iodide and the chemical and antimicrobial stability over the period of about 18 months J Appl Microbiol 2000 89 2 215 224 10.1046/j.1365-2672.2000.01098.x 10971753

[B18] Kariya C Leitner H Min E van Heeckeren C van Heeckeren A Day BJ A role for CFTR in the elevation of glutathione levels in the lung by oral glutathione administration Am J Physiol Lung Cell Mol Physiol 2007 292 6 L1590 1597 10.1152/ajplung.00365.2006 17369290 PMC3983954

[B19] Gould NS White CW Day BJ A role for mitochondrial oxidative stress in sulfur mustard analog 2-chloroethyl ethyl sulfide-induced lung cell injury and antioxidant protection J Pharmacol Exp Ther 2009 328 3 732 739 10.1124/jpet.108.145037 19064720 PMC2682257

[B20] Vesey CJ McAllister H Langford RM A safer method for the measurement of plasma thiocyanate J Anal Toxicol 1999 23 2 134 136 10192420 10.1093/jat/23.2.134

[B21] Hudson VM Rethinking cystic fibrosis pathology: the critical role of abnormal reduced glutathione (GSH) transport caused by CFTR mutation Free Radic Biol Med 2001 30 12 1440 1461 10.1016/S0891-5849(01)00530-5 11390189

[B22] Dawson DC Smith SS Mansoura MK CFTR: mechanism of anion conduction Physiol Rev 1999 79 1 Suppl S47 75 9922376 10.1152/physrev.1999.79.1.S47

[B23] van Dalen CJ Whitehouse MW Winterbourn CC Kettle AJ Thiocyanate and chloride as competing substrates for myeloperoxidase Biochem J 1997 327 Pt 2 487 492 9359420 10.1042/bj3270487 PMC1218820

[B24] Ashby MT Carlson AC Scott MJ Redox buffering of hypochlorous acid by thiocyanate in physiologic fluids J Am Chem Soc 2004 126 49 15976 15977 10.1021/ja0438361 15584727

[B25] Yang J Qu JM Summah H Zhang J Zhu YG Jiang HN Protective effects of imipramine in murine endotoxin-induced acute lung injury Eur J Pharmacol 2010 638 1-3 128 33 10.1016/j.ejphar.2010.04.005 20406626

[B26] Van Der Vliet A Nguyen MN Shigenaga MK Eiserich JP Marelich GP Cross CE Myeloperoxidase and protein oxidation in cystic fibrosis Am J Physiol Lung Cell Mol Physiol 2000 279 3 L537 546 10956629 10.1152/ajplung.2000.279.3.L537

[B27] Guo Y Schneider LA Wangensteen OD HOCl effects on tracheal epithelium: conductance and permeability measurements J Appl Physiol 1995 78 4 1330 1338 7615440 10.1152/jappl.1995.78.4.1330

[B28] Sutherland ER Sputum induction in COPD--it's safe, so now what do we do? Copd 2006 3 2 73 74 10.1080/15412550600651230 17175668

[B29] Toungoussova O Migliori GB Foschino Barbaro MP Esposito LM Dragonieri S Carpagnano GE Salerno FG Neri M Spanevello A Changes in sputum composition during 15 min of sputum induction in healthy subjects and patients with asthma and chronic obstructive pulmonary disease Respir Med 2007 101 7 1543 1548 10.1016/j.rmed.2006.12.009 17258444

[B30] Elkins MR Robinson M Rose BR Harbour C Moriarty CP Marks GB Belousova EG Xuan W Bye PT A controlled trial of long-term inhaled hypertonic saline in patients with cystic fibrosis N Engl J Med 2006 354 3 229 240 10.1056/NEJMoa043900 16421364

[B31] Boucher RC Airway surface dehydration in cystic fibrosis: pathogenesis and therapy Annu Rev Med 2007 58 157 170 10.1146/annurev.med.58.071905.105316 17217330

[B32] Hol WG Lijk LJ Kalk KH The high resolution three-dimensional structure of bovine liver rhodanese Fundam Appl Toxicol 1983 3 5 370 376 10.1016/S0272-0590(83)80007-4 6357922

[B33] Kristensen M Krogholm KS Frederiksen H Bugel SH Rasmussen SE Urinary excretion of total isothiocyanates from cruciferous vegetables shows high dose-response relationship and may be a useful biomarker for isothiocyanate exposure Eur J Nutr 2007 46 7 377 382 10.1007/s00394-007-0676-5 17717627

[B34] Howard M Fischer H Roux J Santos BC Gullans SR Yancey PH Welch WJ Mammalian osmolytes and S-nitrosoglutathione promote Delta F508 cystic fibrosis transmembrane conductance regulator (CFTR) protein maturation and function J Biol Chem 2003 278 37 35159 35167 10.1074/jbc.M301924200 12837761

[B35] Zhang XM Wang XT Yue H Leung SW Thibodeau PH Thomas PJ Guggino SE Organic solutes rescue the functional defect in delta F508 cystic fibrosis transmembrane conductance regulator J Biol Chem 2003 278 51 51232 51242 10.1074/jbc.M309076200 14532265

[B36] Taube C Holz O Mucke M Jorres RA Magnussen H Airway response to inhaled hypertonic saline in patients with moderate to severe chronic obstructive pulmonary disease Am J Respir Crit Care Med 2001 164 10 Pt 1 1810 1815 11734428 10.1164/ajrccm.164.10.2104024

